# Utility of a webinar to educate trainees on UK core surgical training (CST) selection – A cross sectional study and future implications amidst the COVID-19 pandemic

**DOI:** 10.1016/j.amsu.2020.08.054

**Published:** 2020-09-09

**Authors:** Nikhil M. Patel, Apoorva Khajuria, Ankur Khajuria

**Affiliations:** a Core Surgical Trainee Year 2, Department of General Surgery, Buckinghamshire Healthcare NHS Trust, United Kingdom; b Core Surgical Trainee Year 1, Department of General Surgery, University Hospital Coventry and Warwickshire NHS Trust, United Kingdom; c Kellogg College, University of Oxford, United Kingdom; d Department of Surgery and Cancer, Imperial College London, United Kingdom

**Keywords:** Webinar, Education, Surgical training, Covid-19, Technology

## Abstract

**Background:**

The application process for Core Surgical Training (CST) in the United Kingdom (UK) is competitive and hence, careful preparation is required for trainees to obtain their posts of choice. There are multiple resources for preparation for selection including face-to-face courses and online question banks, however there is a paucity of webinars to educate trainees. With the cancellation of such courses due to social distancing restrictions caused by the Covid-19 pandemic, this cross-sectional study aims to evaluate the usefulness of a webinar to educate trainees on CST selection in the UK.

**Materials and methods:**

A free online webinar was held on a single day by a second year core surgical trainee and was attended by 111 junior doctors. Beforehand, all attendees were invited to complete a survey on Google Forms (Google, USA) to ascertain their level of experience with webinars, obtain demographic information and elicit their level of knowledge about CST selection using a 1–5 Likert scale.

**Results:**

Most attendees were in Foundation Year 2 (38.7%) and many had not previously attended a webinar as part of CST application preparation (93.7%). Over half of respondents (55.0%) preferred a webinar over a face-to-face tutorial, appreciating the flexibility, convenience and zero financial cost associated. Many candidates received minimal advice on CST application by their Foundation School (47.7%) and 50.5% of respondents rated their confidence on the application process at ‘3 out of 5.‘

**Conclusion:**

Our study suggests webinars have been underused in preparation for CST applications. Traditional courses and advice from colleagues are more popular ways in which applicants prepare for selection. However, given the degree of uncertainty surrounding the return of face-to-face courses due to the Covid-19 pandemic, preparation for CST application may become increasingly reliant on online materials, which may result in an increased demand for high quality, engaging and informative webinars.

## Highlights

• Core Surgical Training (CST) is the first step of a surgical career in the United Kingdom (UK).
• Entry to the 24 month programme is competitive and there are numerous resources available to help prepare.
• Webinars are currently underused in medical education but are becoming more popular during the Covid-19 pandemic.
• Prospective applicants favour the flexibility with which they can attend a webinar to prepare for CST applications.
• Webinars should be used regularly in preparation for CST applications, if face-to-face courses remain cancelled.

## 1 Introduction

**Fig. 1:**
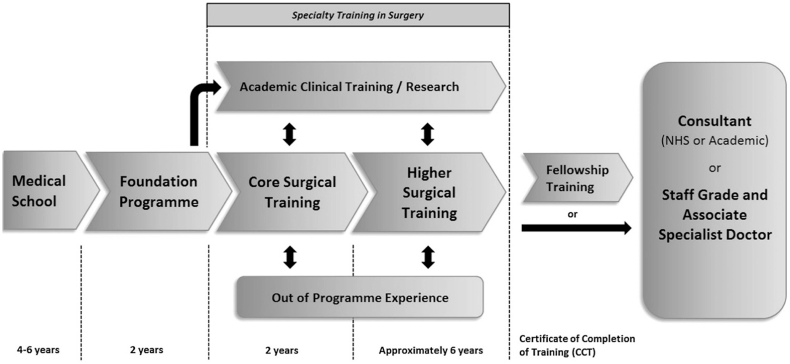
Surgical training pathway in the UK [2].

Core Surgical Training (CST) is the first stage of surgical training in the United Kingdom (UK). The 24-month programme provides the opportunity to experience various surgical specialities and develop operative skills. During this time, trainees are encouraged to identify a speciality for higher training. Fig. 1 illustrates the pathway to becoming a fully accredited surgeon capable of independent practice in the UK. Entry into the CST programme remains a competitive process as highlighted by the yearly increase in competition ratios in Table 1 [1].

**Table 1:** Competition ratios for CST [1].

YEAR	APPLICATIONS	AVAILABLE POSTS	COMPETITION RATIO (APPLICATIONS PER POST)
2016	1622	642	2.53
2017	1608	629	2.56
2018	1870	636	2.94

The increasing size of an ageing and increasingly co-morbid population in the UK has resulted in a rise in the demand for surgeons. We must encourage, inspire and support the next generation of surgeons to accommodate for this population growth and avoid facing problems of a shortage of surgeons as evident in the United Sates (US) [1]. Although a need has been identified for trainee mentorship; there is no national provision for a formal mentorship program in surgical training. Most surgical trainees do not have mentors and report that mentorship for academic and clinical guidance would be of great value [3]. Observational studies have reported the potential for meaningful mentoring via digital technologies [4]. Additionally, there is growing use of digital technologies in education such as virtual learning environments, lecture capture and webinars. Schmidt et al. highlight a lack of mentorship as a key factor influencing students’ decisions to pursue a career in surgery [5].

Mentorship is an important component of career development that transcends the typical hierarchies within the medical profession and enables a senior to guide a less experienced junior in their career [6]. Good mentorship is especially important in inspiring the next generation of surgeons especially as one particular study has highlighted a decline in interest in a surgical career among trainees in the US [7]. This global review on trends of surgical career selection among medical students and graduates identifies factors such as, ‘lack of mentors,’ and ‘negative faculty role models,’ as well as ‘sacrifice of personal time,’ ‘high stress level,’ and ‘inadequate time for family and friends,’ as common negative extrinsic factors dissuading trainees from a career in surgery [7]. Evidence has shown that early exposure to positive role models is integral in attracting and maintaining junior trainees' interest in surgery [7,8].

Social distancing guidelines as a result of the Covid-19 pandemic may have a direct impact on the provision of mentorship which may affect students' decisions to become surgeons. The importance of mentorship in succeeding in a surgical career is highlighted in a study by Rudnicki et al. which, for example, specifically cites mentorship as a vital contributing factor in helping surgeons reach positions of responsibility such as ‘program director’ [9]. Social distancing may therefore see a rise in mentorship through online platforms such as webinars or more informal settings such as conversations over instant messaging, if mentors and trainees are not able to physically meet.

Social media, which is used to provide peer support in the form of interactive groups enabling the sharing of advice may face increasing popularity alongside online video conferencing programmes such as Zoom (Zoom Video Communications Inc. USA) and Microsoft Teams (Microsoft Corporation, USA) to enable the provision of mentorship. A study by Cree-Green et al. has highlighted that informal peer support fosters the success of underrepresented groups within a particular speciality [6]. However, the direct impact of such software on the provision of mentorship is yet to be explored within the context of the Covid-19 pandemic.

Furthermore, social media is increasingly used by organisations to communicate with and educate members of the surgical profession all over the world. A study by Ralston et al. highlighted that the social network Twitter, has enabled the four surgical Royal Colleges in the UK and Ireland, and the American College of Surgeons to reach broader audiences to promote education, training and events by sending over 7000 broadcast messages or ‘tweets,’ in just a 4 year period [10]. Many aspiring and current medical students use Twitter for recreational and educational purposes; therefore regular activity in the form of announcing courses and events aimed at younger students can be used to draw interest from, as well as motivate and inspire the next generation of surgeons [11]. Furthermore, accounts run a livestream of parts of an operation or post photographs of interesting surgical pathology with the intention of generating debate and discussion. On a larger scale, medical schools could use livestream operating as an educational opportunity for students to learn important anatomy and the management of surgical pathology [12]. This may become increasingly popular as the use of virtual reality in the delivery of medical education increases as a result of the Covid-19 pandemic [13].

Webinars have been used regularly by professional bodies and academic institutions as a means of communicating with, transferring knowledge to and facilitating discussion with their members over the internet. They enable a number of delegates to benefit from this form of online seminar by using just an internet connection and a device such as a laptop or tablet computer. Furthermore, they allow more participants to engage and learn than what would be limited by the space within conference venues [14,15]. In essence, they, along with other educational technology help facilitate life-long learning; which is an accepted important aspect of a medical career [16]. Access is granted often by filling out an online sign-up form and then entering a link into a web browser. Viewers may have the opportunity to send in questions either in the weeks leading to the webinar or during the session itself for the session lead to answer. Furthermore, webinars act as a platform to facilitate real-time discussion between the host and the audience, almost identical to the traditional face-to-face seminar.

The current pandemic caused by Covid-19 has resulted in the cancellation of face-to-face surgical courses, seminars and conferences around the world [17]. In response, the delivery of surgical training has been adapted to meet surgeons' needs. This has resulted in learned societies using webinars as a way of keeping their members updated and to share information about the impact of Covid-19 on surgical care and training in the UK. For example, organisations such as the Plastic Surgery Trainees Association (PLASTA) have used this opportunity to run regular webinars to educate trainees on popular topics in the speciality such as, ‘Improving your results in fracture treatment and tendon surgery with WALANT,’ by Dr Don Lalonde, Professor of Plastic Surgery at Dalhouse University [18].

Our group has previously investigated the use of a live webinar as a means of informing medical students about the UK clinical academic training pathway, which has shown to significantly improve participants’ self-rated knowledge and confidence on the Academic Foundation Programme (AFP) application process [19].

This cross-sectional study aims to investigate the usefulness of a free live webinar that we used as a platform to educate prospective applicants preparing for the competitive selection process to CST in the UK.

## 2 Material and methods

The webinar was hosted by AK, a second year core surgical trainee, attended by 111 junior doctors and medical students, and was run for 2 h. This webinar covered the structure of the CST selection interview, portfolio preparation and advice on how to answer clinical knowledge, and management and leadership questions in the respective sections of the interview.

In the three weeks prior to the session, delegates were invited to complete a questionnaire Table 2 on Google Forms (Google, USA) to gauge their level of previous experiences with webinars during undergraduate and postgraduate training. This questionnaire also collected demographic data including stage of training and what other forms of preparation they had undergone for CST selection. Furthermore, they were given the opportunity to send in questions regarding the application process and interview which were answered by the host in a dedicated question and answer component of the webinar. Delegates’ perceptions of the usefulness of a webinar were ascertained using a Likert scale (1 = low, 5 = high) in this questionnaire.

**Table 2:** PRE-WEBINAR questionnaire.

Question	
1. Name	
2. Gender	
3. What is your current post?	1. Pre-Foundation Year 1 (Medical Student) 2. FY1 3. FY2 4. FY3/Clinical Fellow
4. Medical School	
5. Year of graduation from medical school	
6. Are/were you an undergraduate or graduate-entry medical student?	
7. Have you undertaken an additional degree?	1. BSc 2. BMedSci 3. MSc 4. MRes 5. MD 6. PhD/DPhil 7. BDS 8. I do not have an additional degree
What Foundation School are you in?	1. East Anglia 2. Essex, Bedfordshire and Hertfordshire 3. Leicester, Northamptonshire and Rutland 4. North Central and East London 5. North West London 6. North West England 7. Northern 8. Northern Ireland 9. Oxford 10. Peninsula 11. Scotland 12. Severn 13. South Thames 14. Trent 15. Wales 16. Wessex 17. West Midlands (Central, North and South) 18. Yorkshire and Humber
How many webinars for medical education have you attended?	
Have you previously attended a webinar on Core Surgical Training?	1. Yes 2. No
To what extent to you agree or disagree with the statement, ‘webinars offer flexibility and convenience with e.g. not having to spend time and money on travelling?’	Likert Scale 1–5: Strongly disagree to Strongly agree
Would you prefer a face-to-face tutorial or a webinar?	1. Face-to-Face tutorial 2. Webinar
Which, if any, other training programmes are you considering applying to?	1. Cardiothoracic Surgery 2. Neurosurgery 3. Integrated Medical Training 4. Core Anaesthetics 5. Radiology 6. Obstetrics and Gynaecology 7. Core Psychiatry 8. General Practice 9. Paediatrics 10. ACCS: Acute Medicine/Emergency Medicine/Anaesthetics 11. Emergency Medicine 12. Ophthalmology 13. Public Health 14. Community Sexual and Reproductive Health 15. Histopathology 16. Out of programme experience/‘FY3′ 17. Academic Clinical Fellowship (ACF)
How interested are you in applying to Core Surgical Training?	Likert Scale 1–5: Extremely uninterested to Extremely interested
How knowledgeable do you feel about the Core Surgical Training application process?	Likert Scale 1–5: Extremely knowledgeable to Extremely knowledgeable
How confident do you feel about the Core Surgical Training application process?	Likert Scale 1–5: Extremely unconfident to Extremely confident
To what extent has your Foundation School informed you about applying to and undergoing Core Surgical Training?	Likert Scale 1–5: Minimal advice to Extensive advice
What other resources have you used to learn more about the Core Surgical Training application process?	1. Websites 2. Published journal articles 3. Books 4. Courses (not organised by your Foundation School) 5. University (lectures, careers team) 6. Societies e.g. ASiT 7. Friends and colleagues 8. Nothing

This cross-sectional study ascertains delegates’ opinions of a proposed teaching intervention, within the context of their views on various other educational resources available in preparing for CST applications, and therefore, no ethical approval was required. No incentives were offered for completing the pre-course questionnaires and students were reassured that these were purely for the purpose of evaluating the success of the webinar.

Criteria as stated in the 2019 STROCCS guideline were followed in the reporting of this cross-sectional study [20].

## 3 Results

Out of 111 respondents, 43 (38.7%) were in Foundation Year 2 (FY2), 63 (56.8%) identified as male, 92 (82.9%) had studied medicine as an undergraduate, 59 (53.2%) had undertaken an additional degree, 72 (64.9%) had never previously attended a webinar as part of their medical education and 104 (93.7%) had never previously attended a webinar for CST preparation.

Of note, 61 respondents (55.0%) declared preference of a webinar over a face-to-face tutorial and 56 respondents (50.5%) stated strong agreement with the statement that ‘webinars offer flexibility and convenience with e.g. not having to spend time and money on travelling.’ Additionally, 87 (78.4%) had used websites as part of preparing for CST selection and 38 (34.2%) had previously attended courses as part of preparation. Of the other programmes available for application, an Academic Clinical Fellowship was the most popular alternative among delegates (37, 33.3%).

Prior to this webinar, most delegates (54, 48.6%), felt neither extremely unknowledgeable nor extremely knowledgeable about the CST application process and 56 (50.5%) rated their confidence on the CST application process as ‘3 out of 5.’ The majority of delegates (53, 47.7%) also felt that their foundation school had offered ‘minimal advice’ on the application process.

## 4 Discussion

The cancellation of face-to-face courses due to social distancing rules that have been established in response to the Covid-19 pandemic may result in a rise in the provision of webinars to support continued provision of medical training and prepare candidates for CST applications. Previous studies have highlighted that a structured and informative face-to-face course can significantly improve candidates’ knowledge, confidence and preparedness when approaching a competitive selection process [21]. Therefore, at a time when the return to such courses remains unconfirmed, webinars must be able to address the needs of trainees where possible.

In this cross-sectional study, the majority of attendees were in FY2 (38.7%), however a proportion was composed of juniors doctors in an FY3 or Clinical Fellow post (13.5%) i.e. not a formal training programme. This reflects the increasing popularity of taking a year ‘out’ after FY2 as highlighted in a survey of 7168 FY2s in 2015 [22]. This survey highlighted the popularity of a career break with trainees taking up roles in e.g. service posts or as anatomy demonstrators. This year can be used to give post-FY2s additional exposure to surgical specialities, extra time to improve procedural skills and opportunities to strengthen their portfolio while providing them with the time to decide if a surgical career is for them and thereby giving them a chance at securing a CST post the following year at selection. This is reflected in our survey given that the majority of attendees (33.3%) were also considering an FY3 or ‘out of programme experience’ as an alternative to CST.

The majority of attendees (50.5%) felt neither confident nor unconfident about the CST application process and a similar proportion (48.6%) felt similarly about their knowledge of the CST application process as a whole. This highlights the need for an informative webinar to address this gap in their knowledge, and improve their confidence as the interview approaches.

Furthermore, 47.7% of attendees reported receiving minimal advice from their Foundation School about applying to and undergoing CST. Therefore, the lack of confidence and knowledge about the CST application is not surprising. It has been reported that when junior doctors are well informed about a particular career option, e.g. academic training, they are more likely to consider pursuing an academic career [23]. The same can be suggested for a career in surgery as evidenced in a study by Bridgeman et al., in 2016 where a medical student-led extracurricular engagement event managed to significantly improve interest in a career in Cardiothoracic Surgery [24]. A way of initiating action could involve Foundation Schools inviting careers officers at their Local Education Training Board (LETB) to run online events on preparing for speciality applications during protected Foundation Year teaching sessions.

As highlighted by our results, the majority of trainees have not had any experience with webinars as part of their medical training (64.9%), and even fewer as part of preparation for CST applications (4.5%). Traditionally more popular forms of preparation include websites e.g. online question banks and working with colleagues who may be involved in selecting future trainees, such as consultants or current core surgical trainees who have recently been successful at applications. Websites were used by 78.4% of attendees making them the most popular way to prepare. This was confirmed by a survey of 1083 medical students published by Wynter et al. which highlighted that online question banks were among the most popular learning tools as 90.6% of respondents used these for exam revision [25]. Such resources can be informative; explaining to applicants how the application process works, the structure of the interview and how to prepare a portfolio, or they can be a source of ‘mock stations’ replicating the style of questions to help the applicant practice. The candidate can practice in the comfort of their own home where they can take their time to understand the application process and assessment methods in an unhurried manner.

Somewhat unsurprisingly, formal courses remain a popular method of preparation for CST with 34.2% of trainees reporting attending such courses. Such courses provide a combination of didactic sessions explaining the composition of the interviews and common topics to prepare before running mock interviews to allow trainees to practice interview-style questions. By mimicking the CST interview, they remain a popular method of preparation. Furthermore, our survey identified 72.1% of webinar attendees used friends and colleagues as a way of learning about the CST application process; a formal interview course gives them the opportunity of practicing with new people and therefore bring them closer to the real interview where they will be interviewed by consultants they have not worked with before.

Over half of attendees (55.0%) declared preference of a webinar over a face-to-face tutorial despite the majority having not had any webinar experience in their medical education. There are several reasons that may explain this. Firstly, one benefit of webinars over traditional courses is accessibility; a smartphone or laptop with an internet connection grants the trainee immediate access to the webinar without, among other expenses, the cost of transport to and from a particular venue [26]. In support of this, over half of respondents (50.5%) strongly agreed with the statement that ‘webinars offer flexibility and convenience with e.g. not having to spend time and money on travelling.’ Furthermore, the preference of webinars may be explained by the learning patterns of this particular generation of delegates [27].

The use of information technology in medical education has increased significantly over time in response to the challenges it faces [28]. These include the need for lifelong learning, a change in curricular emphasis towards meeting competencies and a new generation of learners who have grown up with technology incorporated from early in their education [28]. Therefore, it can be extrapolated that such a generation would choose easily available electronic learning resources over traditional face-to-face alternatives [29]. A webinar is able to maintain a level of interaction to keep students engaged and interested but can also deliver the information required clearly, with the added convenience of being able to attend the webinar from anywhere with internet access. It therefore comes as no surprise that such junior doctors and medical students who have been exposed to technology throughout their education favour the webinar format due its perceived convenience [29].

Trainees have the opportunity to submit questions before or during the webinar for answer by the host without mental barriers created by nervousness. The ‘live-feed’ of comments that are often part of the webinar can be viewed by the host and attendees; important discussion points may be raised here which enables attendees to get more out of the webinar. Furthermore, they can benefit from hearing the answers to questions posed by other attendees which may also signpost areas to the attendee that need further study. This may improve overall engagement and satisfaction with the webinar.

A webinar removes some potential technological issues which may arise during a traditional face-to-face course. Slideshow presentations are a common component of such courses, and with this come the technical ‘glitches’ that may arise from using a projector and microphone which can cause delays. Such issues are virtually eliminated by a webinar as the host simply uses their own computer to deliver the session and can share their slideshow in real-time with all attendees over the internet.

Overall, there are numerous advantages to webinars in medical education. As well as the accessibility of this learning platform, webinars can be recorded and archived allowing anyone to benefit from the session at a later date [30]. Furthermore, the webinar can be played back for repeated viewing if delegates wish to re-visit an important topic or discussion point. A meta-analysis of 12 randomised controlled trials highlighted the geographical flexibility of webinars as a significant advantage over face-to-face learning, as well as the option of synchronous ‘many-to-many’ interaction which cannot be afforded by other online learning platforms such as pre-recorded lectures and webcasts [31]. This advantage allows for open discussion between the tutor and the students. As well as opportunities for discussion and to ask questions via the ‘chat’ option present in webinar software, audience participation can be encouraged in other ways such as through polls, quizzes and ‘virtual rooms’ for group work as part of a break-out session [31].

Despite the numerous advantages of webinars highlighted in the literature, there are several negative aspects of webinars [12,13,16,19,30,31]. The presenter does not see the listeners, especially if presenting to large groups, as was the case in this webinar. Therefore, it is difficult to tell if the students are following and are engaged or not [32]. The option to record webinars for future reference may also result in poor engagement of students when the webinar is delivered in real-time; they may not engage as much as they know they can watch the webinar again at their own convenience, as opposed to if they were in a seminar room or lecture theatre, surrounded by colleagues without the option if listening to a recording of the session later [33]. Only one participant can be heard at a time; interruptions can be even more disruptive in a webinar therefore a break in conversation is needed for participants to add comments or ask questions [34]. Furthermore, the webinar experience may be detrimentally affected by poor internet reception or WiFi; making it challenging for the presenter to deliver and the delegates to benefit from the session. This can distort the quality of the session, and thereby negatively impact learning. In the middle of a webinar, technical glitches may be difficult to manage immediately and interrupt the natural flow of the session. This together with background noise caused by other listeners running several computer programs at the same time disrupts the learning experience for all [33]. Therefore, although there are many advantages to webinars, they must be delivered and received using the appropriate technological infrastructure to ensure a high-quality learning experience.

Our study is limited by our small sample size, given that this was a single webinar hosted on a single day, and that we did not invite delegates to complete a post-webinar questionnaire. The latter would have provided additional data to perform statistical analysis to determine whether this webinar made trainees feel significantly more prepared for CST applications and whether they felt a webinar was a useful method of preparation. However, there was representation from 25 out of the 33 UK Medical Schools and a further 5 Medical Schools from around the world, and was available at no cost.

Further work can include expanding this into a cohort study to collect data one year post-application to determine how successful attendees were in achieving a CST post, and what they changed, if anything, about their preparation for interview following this webinar.

## 5 Conclusions

The results of this questionnaire suggest that webinars have been underused as an educational tool in preparing applicants for CST applications. The results of our study highlight that traditional courses are the most popular way in which applicants have prepared for CST, followed by taking advice from friends and colleagues and using online resources. This may be because of the opportunity to receive first-hand information on the experience, take part in mock interview practice and receive direct feedback on performance. Furthermore, candidates may find ‘face-to-face’ practice as a more realistic representation of the interview and therefore valued this form of preparation over webinars initially.

With the future of CST applications yet to be determined given the rapidly changing circumstances of the Covid-19 pandemic, the Joint Committee on Surgical Training (JCST) are yet to comment on whether face-to-face interviews will return for the next recruitment cycle [35]. Furthermore, until there is clear consensus on whether face-to-face courses will be permitted, preparation for CST application may be increasingly reliant on the provision of online materials, which may result in an increased demand for high quality, engaging and informative webinars.

## 6 Provenance and peer review

Not commissioned, externally peer-reviewed.

## Conflict of interest statement

The authors have no conflicts of interest to declare.

No funding was required to carry out this research.

## Sources of funding

There were no sources of funding for our research.

## Ethical approval

Ethical approval was not required for this research because this cross-sectional study is an evaluation of a teaching intervention.

## Research registration Unique Identifying Number (UIN)

Name: Research Registry.

Hyperlink: https://www.researchregistry.com/register-now#home/registrationdetails/5ef3d8c2cbc4310016f680da/

Unique Identifying Number (UIN): researchregistry5748.

## Author contribution

Mr Nikhil M. Patel-data collection, data analysis, writing.

Dr Apoorva Khajuria-data collection, data analysis, writing.

Mr Ankur Khajuria-study design.

Nikhil M. Patel and Apoorva Khajuria are both joint first authors in this paper.

## Guarantor

Mr Ankur Khajuria.

Email-ankur.khajuria09@imperial.ac.uk.

## Declaration of competing interest

The authors have no conflicts of interest to declare.
